# Using Gene Expression to Annotate Cardiovascular GWAS Loci

**DOI:** 10.3389/fcvm.2018.00059

**Published:** 2018-06-05

**Authors:** Matthias Heinig

**Affiliations:** ^1^Institute of Computational Biology, Helmholtz Zentrum München German Research Center for Environmental Health, Neuherberg, Germany; ^2^Department of Informatics, Technical University of Munich, Munich, Germany

**Keywords:** eQTL, expression quantitative trait loci, genome wide association study, GWAS, cardiovascular disease

## Abstract

Genetic variants at hundreds of loci associated with cardiovascular phenotypes have been identified by genome wide association studies. Most of these variants are located in intronic or intergenic regions rendering the functional and mechanistic follow up difficult. These non-protein-coding regions harbor regulatory sequences. Thus the study of genetic variants associated with transcription—so called expression quantitative trait loci—has emerged as a promising approach to identify regulatory sequence variants. The genes and pathways they control constitute candidate causal drivers at cardiovascular risk loci. This review provides an overview of the expression quantitative trait loci resources available for cardiovascular genetics research and the most commonly used approaches for candidate gene identification.

## Background

The ultimate goal of any genetic association analysis is to identify genetic variation linked to variation of a phenotype and to elucidate the molecular mechanisms, which are altered by the sequence variation. Genome wide association studies have been tremendously successful in identifying thousands of disease-associated loci as documented by the steady growth of the continuously updated GWAS catalog (1). This progress has also highlighted hundreds of loci associated with cardiovascular phenotypes: the current GWAS catalog (2) lists 249 distinct chromosomal regions associated with coronary artery disease with candidate genes and pathways at many loci summarized in Klarin et al. (3), 138/115 with diastolic/systolic blood pressure, 109 with QT interval, to name just the top three cardiovascular phenotypes. Follow up analysis of these loci aim to establish the causal mechanisms underlying the statistical associations. In classical family based linkage studies typically identifying rare variants with very large effect sizes, the causal variants are typically located in the protein sequence and have a strong impact on protein function (4), for instance truncating mutations in the sarcomeric protein TTN cause dilated cardiomyopathy (5–8). In GWAS however, the identification of causal variants proved to be very challenging, since the vast majority of these disease-associated variants is located either in introns of genes or in intergenic regions (2). Therefore the classical approach of identifying the variant with strongest impact on protein function, such as gained stop codons is not sufficient.

Recent large-scale efforts have annotated a plethora of functional regulatory elements such as enhancers residing in the non-protein-coding part of the genome (9, 10). Therefore an alternative mechanism might be that disease-associated regulatory variants alter the sequence and function of such regulatory elements. Indeed a systematic analysis of the location of disease-associated variants showed that they preferentially reside in regulatory elements (11, 12). Since regulatory elements are highly tissue specific, this information can even be used to identify the disease-relevant tissues (11, 12). These results from localization analysis are highly suggestive that disease-associated variants alter regulatory elements. It now remains to be shown that they indeed are altered and to identify the respective target gene whose transcription is controlled by the regulatory element.

Integrated analysis of the genetics of gene expression provides an elegant way of directly assessing the consequences of putative regulatory sequence variants on transcription. In this study design (13), a population cohort is characterized for their genome wide patterns of genetic variation and also for genome wide gene expression. Gene expression levels are treated as quantitative traits and systematically tested for associations between sequence variants and gene expression. Significant associations are called expression quantitative trait loci (eQTL). These eQTL not only identify putative regulatory variants, but also their target genes as the gene whose expression is associated with the variant (14, 15). Biological information processing and regulation is not limited to transcription, so this approach has also been generalized toward other intermediate molecular traits such as DNA methylation (16, 17), open chromatin (18), histone modifications (19–21), gene, exon and transcript expression levels (22–26) translation and protein levels (27) as well as metabolites (28, 29). In particular the information from the epigenome can be used to identify regulatory variants, and to characterize their role in disease (11, 18, 21, 27).

## eQTL resources for cardiovascular genetics

Regulatory elements and also the effects of variants on those elements can be highly tissue specific, therefore it is key to investigate the tissue relevant for the disease (11, 12, 25, 30). Because biopsies of tissues relevant for cardiovascular diseases, in particular of the heart are very difficult to obtain from humans, it is not surprising, that early applications of eQTL analysis to identify candidate genes for cardiovascular phenotypes were reported in animal models (31). To understand the regulatory impact of sequence variants in humans, samples of disease relevant tissues are often obtained during surgery, from organ donors or from post-mortem sections. As a consequence of these practical considerations, the transcriptome data might be confounded by differences in tissue composition (32) or ischemic time of post-mortem samples (25). Therefore additional care has to be taken in data analysis accounting for observed and hidden confounders (33). Current reviews provide an overview of recent human eQTL studies (15, 34). The most comprehensive study to date is the Genotype tissue expression (GTEx) project, which aims to characterize regulatory sequence variants across 44 distinct tissues from post-mortem sections (26). This includes cardiac tissues: left ventricle, atrial appendage; vascular tissues: aorta, tibial artery, coronary artery; as well as metabolic tissues: liver, subcutaneous and viscelar adipose tissue (Table 1). In terms of sample size and coverage of tissues of interest, the eQTL data generated in the STARNET consortium is currently the most comprehensive resource (38). It focuses on vascular and metabolic tissues in patients with coronary artery disease. It has been shown that eQTL are sometimes dependent on the disease context (32). This observation is also supported by the finding that more eQTLs associated with disease SNP can be found in diseased populations (38). Formation of atherosclerotic plaques is an inflammatory process, therefore also immune cells such as monocytes or macrophages are considered disease relevant tissues and have been extensively profiled (39). Since the disease relevant tissues are not always known a priori efforts are currently underway to establish cohorts of induced pluripotent stem cell that can potentially be differentiated into any cell type for genetic mapping (40). These eQTL projects are complemented by large scale projects aimed at creating a reference map of regulatory elements across an exhaustive set of 111 human cell types and tissues (10) by annotation with epigenetic markers of regulatory elements and recent developments of sequencing based methods (e.g., Hi-C) to study chromosomal architecture (41) in a wide variety of human tissues (42) including heart, liver and aorta. These techniques can identify promoter—enhancer interactions and have already been used successfully to identify IRX3 as the causal gene underlying an obesity GWAS hit located in the intron of the FTO gene (43).

**Table 1:** Recent cardiovascular eQTL resources.

**References**	**Tissue**	**Sample size**	**Population**
(35)	Left Atrial wall	62	European
(32)	Left Ventricle	205	European
(36)	Left Atria	329	European/African American
(37)	Left Ventricle	129	European
(26)	Atrial Appendage	264	European/African American
(26)	Left Ventricle	272	European/African American
(26)	Aorta	267	European/African American
(26)	Tibial artery	388	European/African American
(26)	Coronary artery	152	European/African American
(26)	Adipose—Subcutaneous	385	European/African American
(26)	Adipose—Visceral	313	European/African American
(26)	Liver	153	European/African American
(38)	Mammary artery	600	European
(38)	Atherosclerotic aortic root	600	European
(38)	Visceral abdominal fat	600	European
(38)	Skeletal muscle	600	European
(38)	Liver	600	European

## Candidate identification strategies

### cis eQTL candidate genes

Overlapping eQTL and GWAS SNPs is the most straightforward approach to identify candidate genes for GWAS hits. If a GWAS SNP is also an eQTL for a close by gene or in tight LD with an eQTL, it is conceivable that the SNP indeed affects a regulatory element controlling the expression the respective gene. These genes are typically called *cis*-eQTL when the distance between gene and variant is not further than 500 kb−1 Mb, as opposed to *trans*-eQTL, where the distances are greater or the variant and gene are located on different chromosomes. Cardiovascular candidate genes such as SORT1 (44) and LIPA (45) have been identified as *cis*-eQTL. It has been demonstrated that these candidate genes frequently are not the genes located closest to the GWAS SNP for heart related traits (32) and also more generally for any GWAS trait (25, 26). Nowadays, this candidate annotation approach is becoming a standard analysis included in many GWAS papers and can be performed conveniently using the online software FUMA (46). For instance a recent GWAS on CAD (47) identified eQTL for 196 genes at 97 of the 161 CAD loci found in the analysis from GTEx and other eQTL data bases. This result already demonstrates one caveat of the approach: several candidate genes might emerge for a locus and might be inconsistent between tissues or GWAS variants might also associate with eQTL by chance (26). In this particular example 36 loci have unique candidate genes and additional 24 loci have candidate genes detected consistently across tissues, so 60 loci can be annotated confidently. Overall a highly significant enrichment of trait associated SNPs can be observed among eQTLs as demonstrated for heart related traits (32). Less frequently also trans-eQTL are considered for the annotation of GWAS SNPs, as they do not readily provide a clear mechanistic explanation. Nevertheless, it has been shown in a systematic analysis of GWAS variants, that they frequently also associate with expression levels of genes distant to the GWAS locus (48).

**Figure 1:**
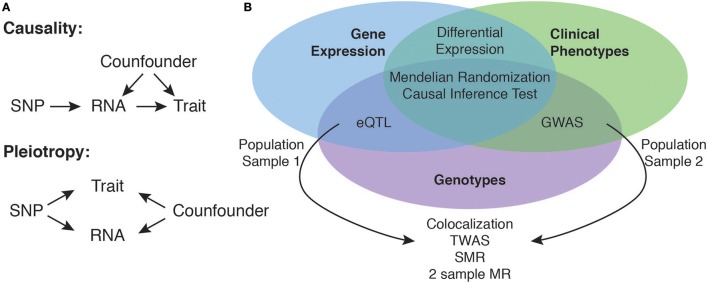
Using eQTL data to identify causal candidate genes at GWAS loci. Integration of eQTL and GWAS data allows for the identification of candidate causal genes, where the effect of the genetic variant (SNP) on the complex trait is mediated by expression levels of an RNA encoded at the locus **(A)**. Overlapping associations of gene expression and clinical trait at the same locus are however not sufficient to infer causality, as they might also be explained as independent pleiotropic effects **(A)**. Depending on the availability of overlapping individual level data sets of genotypes, gene expression and clinical traits there exist several statistical methods to perform causal inference from the data **(B)**.

An important limitation of the overlap-based strategy is that it cannot be used to establish causality. Strictly speaking the experimental design does only allow inferring causality in a statistical sense. In genetic associations the direction of causality is always fixed (Figure 1A). To establish a causal chain between genetic variation, gene expression and the disease phenotype in the strict sense, an interventional experiment would be required, where all other confounding factors that could determine the phenotype are fixed and only the gene expression level would be manipulated to test an effect on the phenotype. If gene expression is indeed causal for the phenotype, any change of the gene expression necessarily would cause a change in the phenotype. In the concept of Mendelian randomization (MR) one is considering a genetic variant as instrumental variable controlling the levels of gene expression and observes its effect on the phenotypic outcome (49). In analogy to randomized control trials, individuals get assigned to a group based on their genotype. Because the direction of causality between genetic variant and gene expression is fixed and the genetic variant is robustly associated with expression levels, one group will receive a higher dose of gene expression. Assuming that the genotype is independent of confounding factors (Figure 1A) changes in phenotypic outcome can be attributed to the changes in gene expression.

Classically, MR and similar approaches to statistically establish causality (50, 51) require to measure all variables in the same population (Figure 1B). This is often not feasible, as gene expression profiling in each and every disease cohort is prohibitively expensive. In practice GWAS SNPs and eQTLs are identified in separate populations. Because of data privacy regulations, often a researcher only has access to the full individual level data of one population and the summary statistics of the other population. Depending on which full data set is available there exist several methods allowing to directly integrate the measured data with summary statistics (52–55). A Bayesian co-localization approach based on summary statistics (56) is testing whether the co-localization of two association signals is compatible with a common underlying causal variant and has been successfully applied to blood lipid traits and liver eQTL. An alternative approach is to impute gene expression levels (57) into a GWAS population (54, 58) using eQTL summary statistics from an eQTL reference population. Subsequently the imputed gene expression can be correlated to the disease phenotype to identify candidate genes (54, 58). Alternatively the transcriptome wide association study (TWAS) method (54) and other methods (Barbeira et al. in review) can also work completely without individual level data by indirectly associating expression and phenotype using eQTL and GWAS summary statistics and the LD structure between SNPs. The TWAS approach showed superior power compared to colocalization analysis and simple overlap based analysis in cases where the causal variants are not directly observed, or when multiple causal variants affecting expression and phenotype exist. Consistent with other candidate identification strategies, analysis of obesity related traits with TWAS showed that 66% of identified trait associated genes were not the closest gene (54). Summary data-based Mendelian Randomization (SMR) is a method that can be used if only summary statistics are available from both eQTL and GWAS results. The method makes use of standard two-sample MR (59) to identify causal or pleiotropic effects of sequence variants on gene expression and phenotypes and distinguishes this situation from overlapping independent causal variants in LD using a test on multiple SNPs (55). Similar to results from TWAS analyses, the application of this method to five common diseases showed that only 60% of the identified candidate genes are the closest gene to the GWAS SNP.

### Network based analysis

Genes are not acting in isolation, but rather form functionally related pathways and networks. Pathways are usually defined based on curated prior knowledge about well-studied processes such as biochemical reactions and signaling pathways (KEGG, Reactome, GO). Pathways can be represented as sets of genes of the same process or as networks preserving the topological information which genes are connected to one another, for instance by catalyzing adjacent steps in a metabolic pathway. Alternatively, networks can be derived from high-throughput experiments such as transcriptome profiling (co-expression network) or protein-protein interaction (PPI) screening (PPI network). Pathways and networks defined either from prior knowledge or from data can subsequently be used for the interpretation of disease associations derived from GWAS. Representing pathways as sets of genes, one can ask, whether a set of genes shows higher evidence of association to disease than random gene sets of the same size. Because GWAS test individual SNPs and not genes, a mapping between SNPs and genes is required, for instance based on genomic positions. Methods such as SNP set enrichment analysis (60, 61) can then be used to test the statistical significance of the association between gene sets and the GWAS results by comparing the distribution of GWAS *P*-values of SNPs within the pathway to a background distribution. These methods have been applied to show the association between CAD and pathways for lipid metabolism, coagulation, immunity (62).

Since eQTL experiments require transcriptome profiling in large cohorts, it is natural to use this data to define data driven gene co-expression networks and gene sets, so called co-expression modules. These gene sets are then annotated according to their gene function or cell type specificity and then related to disease via GWAS results using SNP set enrichment analysis. The link between genes and SNPs can naturally be established via cis-eQTLs of the genes of a co-expression module. This approach was also used in the CAD study mentioned above (62). It is important to note that co-expression modules are not necessarily fully overlapping with biochemical pathways although they might represent the same disease process. For instance the modules might contain transcriptional regulators and parts of a biochemical process that they control.

Network topology of co-expression networks is often used to prioritize candidate genes based on the assumption, that genes with many network connections (so called hubs) are more important (38, 62–65). A study investigating shared molecular networks and their drivers between cardiovascular diseases and type 2 Diabetes applied this strategy (64). Knockout mice for selected key driver genes show indeed metabolic phenotypes and gene expression changes in the network neighborhood of the key drivers. Similarly several studies on CAD identified key driver genes and provided evidence for their functional implication in mouse (65) and *in vitro* studies (62, 65).

## Conclusions

eQTL data provides first leads toward uncovering the mechanisms underlying the statistical associations observed between genetic loci and common cardiovascular diseases. Major challenges for a broad applicability of this approach need to be overcome. First, regulatory elements and therefore also the regulatory impact of sequence variation is highly cell type specific. The GTEx project is addressing this challenge by providing a large scale cross tissue eQTL data base. However, not all conceivable tissues and cell types can be systematically analyzed. In particular transient developmental stages might leave a lasting phenotypic footprint. Induced pluripotent stem cells from cohorts offer an elegant solution (40) as they can potentially be differentiated into any cell type or developmental stage (Nguyen et al. in review) and studied for eQTLs. A second challenge is posed by variability of the genetic effects on expression between different cells making up a tissue and even between cells of the same cell type. eQTL mapping based on single cell transcriptomic data is becoming feasible (66) and can be used to quantify and map the genetic determinants of cell to cell variability of gene expression. Lastly the grand challenge is to move from correlation or co-localization toward causation. Clearly this is the most difficult task and requires on top of rigorous statistical approaches such as MR also experimental validation.

## Author contributions

The author confirms being the sole contributor of this work and approved it for publication.

### Conflict of interest statement

The author declares that the research was conducted in the absence of any commercial or financial relationships that could be construed as a potential conflict of interest.
